# The Neurobiology of Methamphetamine Addiction and the Potential to Reduce Misuse Through Conjugate Vaccines Targeting Toll-Like Receptor 4

**DOI:** 10.7759/cureus.40259

**Published:** 2023-06-11

**Authors:** Savita Prasad, Phoebe S Mathew, Brian J Piper, Karndeep Kaur, Maria Tian

**Affiliations:** 1 Medical Education, Geisinger Commonwealth School of Medicine, Scranton, USA

**Keywords:** neurological methamphetamine effects, clinical methamphetamine effects, methamphetamine use, methamphetamine addiction, methamphetamine vaccines

## Abstract

The methamphetamine epidemic continues to worsen each year and has contributed to more overdose deaths than opioids. Methamphetamine was listed in the top ten lethal drugs in 2021 in the United States. The drug has been shown to cause health problems such as addiction and neurological and behavioral changes. One possible solution to address this crisis is through vaccinations. Vaccinations consist of injecting a controlled substance with the goal of creating compound-specific antibodies. Although still early in development, vaccinations have been found to improve withdrawal symptoms and decrease drug-seeking behavior with minimal health side effects in rodent studies. This paper provides an overview of the clinical presentation and neurobiology of methamphetamine addiction and drug-seeking behaviors. The responses and adverse effects of conjugate vaccines IXTv-100 with adjuvant glucopyranosyl lipid A administered in oil-water stable emulsion and tetanus-toxoid conjugated to succinyl-methamphetamine adsorbed on aluminum hydroxide combined with adjuvant E6020 are examined.

## Introduction and background

This article was previously posted to the Preprints.org server on May 2, 2023.

Methamphetamine was synthesized in 1893 and is a derivative of amphetamine. Over the years, drug applications were found for depression, narcolepsy, weight loss, and as an over-the-counter nasal decongestant [1,2]. The stimulant played an extensive role in keeping troops awake in World War II [2]. With this drug having a high potential for physical dependence, recreational use of the crystalline white powder was outlawed in the US in 1970 [1]. Despite this, misuse dramatically increased in the 1990s [3].

Methamphetamine use disorder is a global concern, impacting users’ organ systems, particularly the neurological and cardiovascular systems [4]. Approximately 35 million people worldwide and 10 million people in the US used methamphetamine in 2020 [5-7]. There are several recreational routes of administration whether it be oral, snorted, smoked, or injected [6,8,9]. This lipophilic agent rapidly crosses the blood-brain barrier, allowing the drug to have an increased absorption in the central nervous system (CNS) and having lasting effects [10]. Reports from the Research and Development Corporation in 2009 showed the economic burden of methamphetamine use in the US was $23.4 billion [11]. These costs include long-term treatment and rehabilitation as well as premature death [11].

There are limited efficacious pharmacotherapeutic options available to combat this highly addictive substance [12]. Immunopharmacotherapy has shown promise as a treatment strategy for drug addiction in recent years [13]. Evidence supporting the use of vaccines for addiction included a significant increase in rodents’ ingestion of cocaine and nicotine to achieve similar effects as unvaccinated baseline levels [14]. Given the findings of the cocaine and nicotine studies, the development of a vaccine for methamphetamine has the potential to treat methamphetamine misuse [13,14].

The advantage of using vaccines include expectations of high patient adherence rates, and given treatment consists of a few injections over a brief period of time compared to taking pharmaceutical treatments daily or on a weekly basis to produce active immunization, reduction of adverse effects, reduction of drug reinforcement, and prevention of overdoses and relapses during recovery [15].

Herein, we reviewed the literature on the clinical presentation of methamphetamine misuse, the neuropsychiatric drug-seeking behavior, the responses of anti-methamphetamine vaccines IXTv-100 with adjuvant glucopyranosyl lipid A administered in oil-water stable emulsion (GLA-SE) and tetanus-toxoid conjugated to succinyl-methamphetamine (TT-SMA) adsorbed on aluminum hydroxide (alum) combined with adjuvant E6020, and the adverse effects in vaccinated rodents.

## Review

Methods

Literature reviews on vaccine treatments for drug misuse, as well as the effects of methamphetamine, were conducted using a variety of sources and credible databases such as PubMed and Google Scholar using search terms: “methamphetamine,” “methamphetamine addiction,” “neurological methamphetamine effects,” “clinical methamphetamine effects,” and “methamphetamine vaccine.” Research articles were deemed valid and credible for involvement as they were published in peer-reviewed journals and sourced from respectable databases. The search was limited to articles published in English with no restrictions on the publication dates on the neurobiology of methamphetamine misuse and the clinical presentation. Articles that evaluated preclinical investigations on the reduction of methamphetamine misuse with the use of conjugate vaccines targeting toll-like receptor 4 ranged from 2013 to 2022.

Clinical presentation of methamphetamine addiction

Methamphetamine has a high lipid solubility that leads to rapid transfer of the drug across the blood-brain barrier [16-18]. Methamphetamine targets the CNS via sympathomimetic action. After ingestion, physiological changes such as increased blood pressure, increased temperature, and pupil dilation occur by reverse transport to redistribute catecholamines [16,17]. Other effects of methamphetamine include autonomic instability such as increased heart rate, bronchodilation, hyperglycemia, feelings of euphoria, increased energy levels, alertness, and decreased anxiety [8]. A common symptom associated with chronic methamphetamine misuse is methamphetamine-associated psychosis and hallucinations [19-21]. Side effects of methamphetamine can be short- or long-term [22,23]. When young adults use methamphetamine, the exposure leads to damaged monoaminergic neurons [24-26]. A study investigating young mice found that there was a decrease in motor activity, nervous system activity, and increased behavior problems [24,26,27].

Toxic high doses of methamphetamine have a severe effect on the cardiovascular system such as vasoconstriction, pulmonary hypertension, atherosclerotic plaque formation, arrhythmias, and heart failure [4,28]. A common illness seen with chronic misuse of methamphetamine can be associated with ventricular hypertrophy which predisposes users to methamphetamine-induced myocardial ischemia [22]. The cardiovascular system has complications due to the increase of free radicals that damage cardiac tissues. If methamphetamine is taken intravenously, an internalized accumulation in the lungs leads to increased reactive oxygen species production [4]. Increased reactive oxygen species levels lead to lung damage [22].

Methamphetamine affects the sympathetic and parasympathetic nervous system that regulates the GI [24,27,28]. After a single dose, methamphetamine stays in the human body for almost seven days [25]. The half-life for methamphetamine is 9-24 hours [29]. The effects are seen in the mesenteric vessels within the gut that can lead to acute existential ischemia [27]. Other health problems induced by methamphetamine misuse include vasoconstriction, bowel ischemia, stomach cramping, constipation, and tissue dehydration [27]. GI and gut pain can also increase irritability in misusers. Observed side effects seen in individuals with methamphetamine addiction or withdrawal are weight loss and insomnia [24].

With methamphetamine addiction, physiological and psychological changes take place. An investigation looked at the effects of face asymmetry with methamphetamine addiction and found chronic misuse increased poor performance on face recognition and changes to the individual's face [30]. Another study examined facial appearance changes in methamphetamine users and found the appearance of accelerated biological aging [30]. The following were changes seen to the face with methamphetamine effects: teeth decay, dark circles under the eyes, runny nose, swelling, skin sores, itching, loss of soft tissue, and weight loss [30].

The social impacts of misusing methamphetamine have been examined in children under 18. If misused when young, school attendance decreased in children. The Ontario Student Drug Use Survey 2005 found that in grades 7-12, there were 2.2% of students admitted to using methamphetamine [24]. This study followed children for 8 years and observed that social side effects include sexual promiscuity, weight loss, poor nutrition, and decreased coping strategies [24].

Addiction and methamphetamine misuse have behavioral effects on the individual's social well-being. Not only is there an increase in irritability, but other behavioral patterns are seen. These behaviors are noted in different age groups as well as overall addiction to the drug. Common behavior changes due to addiction and withdrawal include aggression that leads to violent and criminal behaviors [31]. The increase in usage and dependence is seen in drug abuse which can increase addictive behavior. There were social behavioral changes seen in those who use methamphetamine as people adapted to smoking the substance instead of injection when they were around those who do not inject [9,32]. There was a risk of poor standard of living owing to a decline in health and loss of social bonds due to increasing criminal participation related to methamphetamine [32]. Other effects were an increase in sex drive and increased self-confidence [33]. The increase in sex drive as an effect of the drug also contributes to increased HIV rates in misusers [33]. Overall, the impact of drug abuse and methamphetamine addiction is seen in an individual's lifestyle as well as their social behavioral changes.

The neuropsychiatric substrates of drug-seeking behavior

The brain plays a key role in drug-seeking behavior as it is the control center for positive and negative feedback. Sections of the brain work together to stimulate drug-seeking behaviors. While the brain exhibits neuroplasticity and other mechanisms to restore itself, certain brain transformations can increase drug-seeking behaviors, while other areas of the brain suffer irreversible damage [2]. Nearly one-third of methamphetamine users develop psychotic symptoms such as paranoia, auditory and visual hallucinations, persecutory delusions, and an increased risk of suicide and violence [32]. This is important to understand because the brain can increase drug-seeking behavior and induce lasting effects on itself and the body. The information presented on patterns of addiction and behavioral changes may help shape therapy programs.

Some people may be more vulnerable to the effects of drugs due to genetic predisposition, environmental factors, and lifestyle. Genetic makeup contributes to a person’s vulnerability to drug addiction, including environmental impact on gene function and expression [34]. Environmental factors can create lasting epigenetic changes [34]. Conditions such as increased stress and anxiety along with drug usage can result in long-term changes to the brain [34].

Mesolimbic Dopamine System

The mesolimbic dopamine (DA) system is also known as the reward center. It is responsible for euphoric feelings due to the release of neurotransmitters such as norepinephrine and dopamine in the brain [35]. The DA system acts as positive feedback for drug-seeking behaviors which motivates drug use. Rats that were administered a dopamine (D3) receptor antagonist, VK4-116, showed reduced drug-seeking behaviors after drug reinstatement following withdrawal [36]. In another study, rats repeatedly exposed to cocaine (stimulating dopamine release in the brain like methamphetamine) for 24-hour intervals showed an escalation in self-administration and drug intake than when drugs were administered between ten-day intervals [37]. With prolonged and frequent drug usage, both D1 and D2 dopamine receptors in the brain began to decrease. This indicates more dopamine must be released and bind to dopamine receptors in order to achieve prior levels of euphoria. Subsequently, other activities that produce enjoyment such as eating and socializing with family cannot supply enough dopamine anymore to satisfy emotional stimulation, which further encourages drug intake to fill the gap of the “feel good feeling” that dopamine supplies.

Additionally, prolonged drug usage leads to decreases in dopamine transporters [38]. Dopamine transporters are specialized cells in the CNS that remove dopamine from the synaptic cleft [29]. A decrease in dopamine transporters increases dopamine in the synaptic cleft, allowing more dopamine to bind to receptors on the postsynaptic neuron to prolong euphoric effects. Prolonged drug use has also been linked to dopaminergic axon degeneration where severe degeneration has been linked to the development of Parkinson’s disease [29]. While Parkinsonism may be caused by other neurological abnormalities, methamphetamine use has been found as a risk factor for the development of Parkinson's disease and Parkinsonism [29]. There are three core regions in the brain that largely affect drug usage: basal ganglia, extended amygdala, and prefrontal cortex [39].

Basal Ganglia

The basal ganglia are part of the nigrostriatal dopamine pathway that works together with the DA system to produce euphoric effects and increased addiction behaviors. The basal ganglia are responsible for coordinated movements and motivating behavior. It is essential to encourage prolonged drug usage. There are two sub-regions in the basal ganglia that particularly contribute to drug-seeking behaviors. The first region in the basal ganglia is the nucleus accumbens, which is involved in the euphoric experience and positive feedback [35,40]. The second region is the dorsal striatum, which is involved in habituation and other drug-seeking behaviors such as compulsive drug use [35]. In methamphetamine users, the basal ganglia have been linked to structural changes and metabolite alterations associated with the production and persistence of psychiatric symptoms in abstinent users [41]. Rats treated with a neurotoxic regimen of methamphetamine exhibited long-term changes to the striatonigral region when tested three weeks following methamphetamine administration [42]. This data indicates altered basal ganglia function. Increased iron levels typically observed in aging were found in the basal ganglia after methamphetamine use. The basal ganglia are, thus, vulnerable to oxidative stress [43].

Extended Amygdala

The extended amygdala consists of the bed nucleus of the stria terminalis, sublenticular substantia innominata, and the central medial amygdala and controls feelings of stress, anxiety, and unease [44]. It is responsible for the withdrawal effects, also known as the “dark side” of addiction, and is the main reason for drug relapse. With prolonged drug use, addicts transition from seeking rewarding effects to avoiding the emotional distress and physical discomfort that comes with withdrawal [44]. Additionally, the extended amygdala increases sensitivity. This causes “drug hunger” which contributes to compulsive drug-seeking behaviors and leads to habituation to decrease stress. With increased withdrawal effects, the brain sends corticotropin-releasing factor, norepinephrine, and dynorphins into the extended amygdala which contributes to the negative emotional states [45]. Patients who have obsessive-compulsive disorder were more motivated by the desire to avoid stress and negative emotional states compared to controls [46]. This indicates negative reinforcement avoidance plays a role in compulsive behaviors.

Prefrontal Cortex

The prefrontal cortex (PFC) is located in the frontal lobe of the brain and is responsible for executive functions such as planning, solving problems, and making decisions. It plays a role in executing compulsive activities as well as impulse control [35]. With prolonged drug use, the PFC becomes more dysfunctional [35]. Functional magnetic resonance imaging identified numerous metabolic differences in the PFC among methamphetamine users during cognitive and socioemotional tasks [47]. This lowered the ability to make decisions and exert control over their actions in the user which increased risk-taking and lowered self-control.

What PET Imaging Has Shown Us

Positron emission tomography (PET) is most widely used to image the brain in adults with addiction [46]. This technique is minimally invasive and yields information on acute and long-term drug-induced structural and functional changes in the brain over time, making it favorable to study drug addiction [48]. In a study that tracked methamphetamine users through PET imaging, dopamine transporters decreased after repeated methamphetamine use but could be restored after fourteen months of abstinence [36]. This report confirmed structural abnormalities in the orbitofrontal cortex (OFC), anterior cingulate cortex (ACC), and prefrontal cortex (PFC) with hypoactivity in ACC and PFC which supports the diminished control of an addict’s drug usage, make decisions, execute abstinence, and carry out other executive functions [49,50].

Neuroimaging also revealed changes to the extended amygdala. Prolonged use of drugs increased activation of the amygdala and decreased control of the PFC over the amygdala [35]. This decreased control over one’s emotions and stress which increased negative reinforcements. Imaging also showed changes in the metabolic activity in other brain regions involved in drug hunger craving including the thalamus, cerebellum, and striatal complex [35]. Prior to craving sensations, neuroimaging revealed the activation of the ACC which corresponds to the mood states involved in drug need [35]. Through imaging and brain measures, a positive correlation was found between cognitive performance and brain regional volume/density, blood flow, glucose metabolism, creativity, and activation in methamphetamine users [51].

Lastly, drug abuse can harm the neurodevelopment of fetuses. Fetal brain structures, in particular the frontal cortex and subcortical areas, were found to be highly susceptible to functional and structural change due to intrauterine drug exposure [52].

Treatment vaccine response in rodents

Currently, the US FDA does not have any approved medications for the treatment of methamphetamine addiction [14]. Addressing methamphetamine drug-seeking behaviors is difficult given the high drop-out rates in human studies [14]. As of May 2023, there are limited studies on the effects of methamphetamine reduction related to vaccines in humans. However, the reduction of methamphetamine misuse and its relationship to specific vaccines has ongoing research among rodents [13]. Safe and efficacious vaccines need to produce high antibody levels in response to methamphetamine after administration in rodents before progression to human trials. In conjugate vaccines, methamphetamine-like molecules (haptens) are attached to a carrier protein that helps stimulate an immune response. On its own, methamphetamine is unable to produce an immune system response because of its small size [13,15,53]. The immune response recognizes the hapten as foreign and produces antibodies against it. The conjugate vaccine enhances the immunogenicity of methamphetamine. The choice of carrier proteins depends on factors such as safety, immunogenicity, and compatibility with the hapten [14,15,53-55]. Adjuvants are optional substances added to enhance the immune response to the hapten-carrier complex. Adjuvants improve the effectiveness of the vaccine by boosting the immune system's response with an increase in antibody production and providing stronger and long-lasting immunity [14,53]. Using a combination of adjuvants will produce a further robust response [14,15,53]. There are two conjugate vaccines that show promise based on results from rodent studies: IXTv-100 with adjuvant GLA-SE and TT-SMA adsorbed on aluminum hydroxide (alum) combined with adjuvant E6020. These conjugate vaccines target toll-like receptor-4 (TLR-4) to regulate the necessary robust immune responses to antigens [13,56]. TLR-4 is the receptor for lipopolysaccharides in gram-negative bacteria. TLR-4 agonism is required for a sufficient antibody response [13,56].

IXTv-100 With Adjuvant GLA-SE Rodent-Related Studies

Prior studies were conducted to produce methamphetamine antibodies to reduce related addictive behavior with the administration of the vaccine IXTv-100 in rodents [13]. IXTv-100 was formerly known as the conjugate vaccine ICKLH-SMO9 [13,57]. Succinyl-methamphetamine hapten, HSMO9, is covalently bonded to immunocyanon (IC) [6,13,15,57]. Carrier protein keyhole limpet hemocyanin (KLH) previously showed substantial antibody titers with affinity to methamphetamine [6,13,15]. IC and KLH are ingredients used to generate antibodies [6,13,15]. Studies show without an adjuvant, IXTv-100 produced a large variation in response and less than the optimal affinity for the drug [56,57].

The mice were intravenously self-injecting 0.06 mg/kg/infusion of methamphetamine through an indwelling jugular catheter [13]. After the catheter was inserted, the rats needed one week to recover before self-administering training. The infusion was delivered in 200 μL 0.9% heparinized NaCl over 5.6 seconds [13]. The vaccine consisted of 100 μg of IXT-v100 adjuvanted with 5μg glucopyranosyl lipid A stable emulsion (GLA) in 2% oil-in-water stable emulsion (GLA-SE) [13]. GLA is a synthetic TLR-4 agonist. When GLA is administered in an oil-in-water stable emulsion, the antibody response is enhanced [13]. The injection delivered 100μL of the vaccine intramuscularly into the caudal thigh [13]. The mice self-administered methamphetamine over an eight-week period through a reinforcement lever of various dosages before administering repeated injections of IXT-v100 GLA-SE. IXT-v100 GLA-SE decreased levels of methamphetamine-seeking behavior in rodents [13,58]. 

The IXT-v100 GLA-SE vaccine reduced meth-self administration by 50% compared to the rodents' baseline [59]. GLA-SE induced a higher response than aluminum-based adjuvants such as Alhydrogel [12,53,56,57,59]. Anti-methamphetamine antibodies resulting from vaccine administration led to an increase of methamphetamine bound in the peripheral bloodstream and a reduction of drug-seeking behaviors [13,53]. The presence of methamphetamine antibodies decreased circulating methamphetamine levels in the brain. This treatment reduces methamphetamine in the bloodstream to prevent the crossing of the blood-brain barrier [13].

IXT-v100-related vaccines resulted in decreased neurotransmitter release [15]. Methamphetamine usage is known to affect multiple neurotransmitters including dopamine, norepinephrine, and serotonin [15]. By altering the neuropsychological effects caused by methamphetamine misuse, drug-seeking behavior can be reduced [15]. These studies show the benefit of further exploring IXT-v100 as a future vaccine alternative for humans to reduce methamphetamine misuse [13,15].

TT-SMA With Alum and Adjuvant E6020 Rodent Studies

While KLH is an efficient carrier protein, the large size of the protein makes it difficult to control the quality of the haptenation process [6,14,15,54]. Tetanus-toxoid (TT) is a smaller protein in size that makes it easier to manufacture vaccines but produces a weaker immune response compared to the conjugation of SMA and KLH [6,14,53]. An adjuvant is needed to produce a stronger immune response [14]. Prior anti-methamphetamine vaccination studies show only using an alum adjuvant resulted in insufficient antibody production [53,60,61]. Studies were conducted to attenuate methamphetamine-induced responses with the administration of a tetanus-toxoid conjugated to succinyl-methamphetamine (TT-SMA) adsorbed on alum combined with adjuvant E6020 [53,57,60]. E6020 is a TLR-4 agonist. E6020 is a synthetic, water-soluble, lipopolysaccharide phospholipid A dimer from gram negative-bacteria [53,60,61]. This agonist enhances IgG production. The vaccine was prepared using 32μg of TT-SMA conjugate, 3μg E6020, and 1,500μg alum in PBS [61].

A prior study investigated the effects of TT-SMA with alum and adjuvant E6020. The aim of the study was to determine the efficacy of the vaccine by measuring the levels and affinity of anti-methamphetamine antibodies produced as well as the ability to attenuate methamphetamine-induced locomotor responses and the assessment of methamphetamine levels in the brain. Methamphetamine hydrochloride was dissolved in sterile saline based on concentrations of 0.5mg/ml [62]. The mice that were placed in the vaccine treatment group received 2.0mg/kg doses of the drug [62]. The control group received the same volume of sterile saline [62]. The injections were given subcutaneously on weeks 0, 3, and 6 for locomotor testing and intraperitoneally on week 10 to measure the levels of methamphetamine in the blood and brain of the mice in the treatment group [62]. Both groups of mice were habituated to the locomotor apparatus for three days before they began the methamphetamine drug challenge. The control group of mice that did not receive methamphetamine hydrochloride was compared to rodents treated with the E6020 vaccine. The vaccinated rodents had higher rates of antibodies and decreased levels of methamphetamine in the brain compared to the control group [60,62]. A high level of specific antibodies was produced and was bound to methamphetamine to prevent the drug from crossing the blood-brain barrier [62,63]. This mouse study provided further insight into generating human-related vaccines [62].

While adjuvant E6020 was used in vaccines to increase immunization of antigen-specific antibodies, there was a reduction in drug hunger and locomotor-induced responses [61]. More research is needed to uncover the relationship between methamphetamine, drug hunger, and the compulsive need for more methamphetamine [61].

Active Immunity on the Reward System

The effects of methamphetamine misuse on the reward system and on the active immune system are not fully understood in humans [61]. The drug engages the reward system to produce feelings of euphoria and self-confidence that leads to addiction [64]. Methamphetamine activates the reward system after crossing the blood-brain barrier to increase dopamine in the mesolimbic and mesocortical pathways [3]. Methamphetamine increases dopamine, serotonin, and epinephrine [55,64,65]. When methamphetamine is misused, the rapid release of these neurotransmitters affects the reward system [6]. This builds tolerance in the reward system and misusers need higher doses of methamphetamine to attain the euphoric feeling [53]. T cells, natural killer cells, macrophages, dendritic cells, and B cells are affected in humans misusing methamphetamine [66]. A decrease in addiction was found to lead to a decrease in dendritic cells [67].

The goal of active immunization is to create an immunological response against the drug and create an immunological memory with reexposure to the vaccine through booster injection [61]. The immune system is primed to produce an IgG-mediated antibody response to clear the drug out of the periphery when it encounters the substance in the body [61]. TLR-4 is expressed on immune cells such as granulocytes, monocytes, macrophages, and endothelial cells in organs that have circulating B cells [61]. While the role of TLR-4 in regulating the adjuvant enhancement of humoral responses is unknown, it has been determined that TLR-4 agonism is required for a sufficient antibody response [13,61,56].

Rodent studies show further research is needed to create conjugate vaccines [3,6]. Increasing antibodies within the bloodstream reduces the side effects of methamphetamine and decreases the effects on the reward system [55]. By preventing methamphetamine from entering the CNS through vaccines, there might be a reduction in methamphetamine misuse behaviors [3].

Rodent research has found that methamphetamine vaccines can generate a sufficient level of antibodies [22]. However, there are variations in antibody responses [22]. Further study is needed to create a vaccine to help assist the immune system to mitigate the effects of methamphetamine misuse [66].

Adverse Effects 

Past research in rodents showed active immunization using conjugated vaccines can reduce the physiological and behavioral effects of methamphetamine [3,10,12-15,58,68]. IXTv-100 GLA-SE and TT-SMA with alum and adjuvant E6020 were safe, had an affinity for the drug, and produced an immune response [13,59-62]. Active vaccination attenuated methamphetamine-induced disruptions of thermoregulation, wheel running activity, and stereotyped behaviors after methamphetamine was administered in high doses [3,6,59].

Methamphetamine use increased rectal temperature in rodents at 27ᵒC and decreased body temperature at 23ᵒC in unvaccinated control groups [3,6,55]. In vaccinated groups, these disruptions were attenuated [3,6,57,59]. The efficacy of this approach depended on the antibody concentration responses and the affinity and specificity of antibodies to methamphetamine [3]. Methamphetamine hapten (MH6) has created sustainable antibodies that are effective for methamphetamine [6,57,59]. Previous studies have reported MH6-KLH vaccinated groups showed increased >6,600% methamphetamine levels in serum and >60% decreased methamphetamine levels in the brain after injection with the drug [3,55]. This indicates the vaccine offers neuroprotection in the CNS against the effects of the drug [3,55].

Behavior was measured using wheel activity. Given methamphetamine is a stimulant, there was a significant increase in induced physical activity [1]. In both IXTv-100 GLA-SE and TT-SMA with alum and adjuvant E6020 vaccinated rodents, there was a 50-70% decrease in meth-induced wheel activity and locomotor activity [3,53,59,66]. This is consistent with previous stimulant studies with the cocaine and nicotine vaccines [46].

A group of unvaccinated rodents had reduced food intake compared to the rodents given a dose of the IXTv-100 vaccine after high levels of methamphetamine administration [6,12,57,59]. Food habits were not altered in the vaccinated group, indicating the vaccine was successful in preventing methamphetamine-induced impairment of food responses [6,12,59]. The antibodies of the methamphetamine IXTv-100 conjugate vaccine reduced methamphetamine-related behaviors in rodents, showing its usefulness in future pharmacovigilance in humans [12].

Prior preclinical studies using rodents suggest vaccine administration is safe as there were no adverse effects [3,6,12,63]. Vaccination strategies against methamphetamines are still under investigation by researchers and clinicians [69]. Research on hapten design, the chemical position of a linker between the target antigen and carrier protein, modification and selection of carrier proteins, and selection of adjuvant to augment immune response is needed [6,15,53,54].

Strategies utilizing either active immunization have shown potential [69]. Not only can the vaccine efficacy be drastically different in human patients compared to animal models, but also the quantity of antibodies produced and the safety profile including side effects could also be significantly different [69]. Progression to clinical trials using human subjects will provide further information on the safety profile of the vaccine [69].

There are currently no clinical trials given a high drop-out rate [57]. Results from preclinical studies using rodents may not translate to real-life situations humans face. People misusing methamphetamine often combine multiple opioid substances, contributing to increased rates of mortality and morbidity. Human subjects in clinical trials should have a strong motivation to end their drug dependency as there is a risk of overdose to compensate for the vaccine [70]. Because the vaccine limits methamphetamine from crossing the blood-brain barrier, the positive reinforcement of feeling euphoric will not be achieved [70]. Patients who are psychologically dependent on the drug may take higher doses of the drug to achieve a high, putting their lives at risk [70,71].

## Conclusions

These studies showed that a significant decrease in drug self-administration can be achieved via higher levels of a drug-specific antibody to block the drug from entering the brain’s reward center. A sufficient antibody response is required for the immunotherapeutic strategy to be efficacious. Generating methamphetamine-specific antibodies to bind and form complexes with drug molecules in the peripheral bloodstream through the immunotherapeutic strategy resulted in the reduced entrance to the blood-brain barrier and decreased drug effect on the CNS. Effective processing and leukocyte recognition of antibody-drug complexes produced long-lasting antibody responses. As a result, the likelihood of methamphetamine misuse, relapse, and overdose can be potentially reduced. Methamphetamine-conjugated vaccines such as IXTv-100 GLA-SE and TT-SMA with adjuvant E6020 were found to reduce side effects such as hyperlocomotion, impaired food responses, and promotion of thermoregulation.

More clinical trials evaluating the immunological response of vaccines on methamphetamine misuse are needed. The immunological response of conjugate vaccines depends on the hapten design, the chemical position of a linker between the target antigen and carrier protein, and the modification and selection of carrier proteins and adjuvants. The methamphetamine overdose epidemic poses a challenge to public health and places a costly burden on the healthcare system. Given the minimal development of adverse effects and the efficacy of antibodies to reduce drug effects, reducing methamphetamine abuse through vaccinations in humans may be a feasible and promising option in the future.

## References

[1] (2023). Methamphetamine DrugFacts. National Institute on Drug Abuse.

[2] Miller DR, Bu M, Gopinath A, Martinez LR, Khoshbouei H (2021). Methamphetamine dysregulation of the central nervous system and peripheral immunity. J Pharmacol Exp Ther.

[3] Miller ML, Moreno AY, Aarde SM (2013). A methamphetamine vaccine attenuates methamphetamine-induced disruptions in thermoregulation and activity in rats. Biol Psychiatry.

[4] Kevil CG, Goeders NE, Woolard MD (2019). Methamphetamine use and cardiovascular disease. Arterioscler Thromb Vasc Biol.

[5] Salamanca SA, Sorrentino EE, Nosanchuk JD, Martinez LR (2014). Impact of methamphetamine on infection and immunity. Front Neurosci.

[6] Hossain MK, Davidson M, Kypreos E, Feehan J, Muir JA, Nurgali K, Apostolopoulos V (2022). Immunotherapies for the treatment of drug addiction. Vaccines (Basel).

[7] Kiyokawa M, Cape M, Streltzer J (2021). Insights in public health: methamphetamine use during COVID-19 in Hawai'i. Hawaii J Health Soc Welf.

[8] Rusyniak DE (2013). Neurologic manifestations of chronic methamphetamine abuse. Psychiatr Clin North Am.

[9] McKetin R, Sutherland R, Peacock A, Farrell M, Degenhardt L (2021). Patterns of smoking and injecting methamphetamine and their association with health and social outcomes. Drug Alcohol Rev.

[10] Kosten T, Domingo C, Orson F, Kinsey B (2014). Vaccines against stimulants: cocaine and MA. Br J Clin Pharmacol.

[11] Courtney KE, Ray LA (2014). Methamphetamine: an update on epidemiology, pharmacology, clinical phenomenology, and treatment literature. Drug Alcohol Depend.

[12] Rüedi-Bettschen D, Wood SL, Gunnell MG (2013). Vaccination protects rats from methamphetamine-induced impairment of behavioral responding for food. Vaccine.

[13] Keller CM, Spence AL, Stevens MW, Owens SM, Guerin GF, Goeders NE (2020). Effects of a methamphetamine vaccine, IXT-v100, on methamphetamine-related behaviors. Psychopharmacology (Berl).

[14] Shen XY, Kosten TA, Lopez AY, Kinsey BM, Kosten TR, Orson FM (2013). A vaccine against methamphetamine attenuates its behavioral effects in mice. Drug Alcohol Depend.

[15] Lee JC, Janda KD (2021). Immunopharmacotherapeutic advancements in addressing methamphetamine abuse. RSC Chem Biol.

[16] Petit A, Karila L, Chalmin F (2012). Methamphetamine addiction: a review of the literature. J Addic Res Ther.

[17] (2023). National Institute on Drug Abuse. What are the long-term effects of methamphetamine misuse? National Institute on Drug Abuse. https://nida.nih.gov/publications/research-reports/methamphetamine/what-are-long-term-effects-methamphetamine-misuse.

[18] Barr AM, Panenka WJ, MacEwan GW, Thornton AE, Lang DJ, Honer WG, Lecomte T (2006). The need for speed: an update on methamphetamine addiction. J Psychiatry Neurosci.

[19] Chiang M, Lombardi D, Du J (2019). Methamphetamine-associated psychosis: clinical presentation, biological basis, and treatment options. Hum Psychopharmacol.

[20] Carfora A, Cassandro P, Feola A, La Sala F, Petrella R, Borriello R (2018). Ethical implications in vaccine pharmacotherapy for treatment and prevention of drug of abuse dependence. J Bioeth Inq.

[21] Substance Abuse and Mental Health Services Administration (2016). Impact of the DSM-IV to DSM-5 changes on the national survey on drug use and health. https://www.ncbi.nlm.nih.gov/books/NBK519697/.

[22] Darke S, Kaye S, McKetin R, Duflou J (2008). Major physical and psychological harms of methamphetamine use. Drug Alcohol Rev.

[23] Yasaei R, Saadabadi A (2023). Methamphetamine. https://pubmed.ncbi.nlm.nih.gov/30570977/.

[24] Buxton JA, Dove NA (2008). The burden and management of crystal meth use. CMAJ.

[25] Marshall JF, O'Dell SJ (2012). Methamphetamine influences on brain and behavior: unsafe at any speed?. Trends Neurosci.

[26] Boger HA, Middaugh LD, Patrick KS (2007). Long-term consequences of methamphetamine exposure in young adults are exacerbated in glial cell line-derived neurotrophic factor heterozygous mice. J Neurosci.

[27] Prakash MD, Tangalakis K, Antonipillai J, Stojanovska L, Nurgali K, Apostolopoulos V (2017). Methamphetamine: effects on the brain, gut and immune system. Pharmacol Res.

[28] Edinoff AN, Kaufman SE, Green KM (2022). Methamphetamine use: a narrative review of adverse effects and related toxicities. Health Psychol Res.

[29] American Addiction Centers (2023). Effects of crystal meth on the brain and central nervous system. https://americanaddictioncenters.org/meth-treatment/effects-on-the-brain-and-cns.

[30] Harastani M, Benterkia A, Zadeh FM, Nait-Ali A (2020). Methamphetamine drug abuse and addiction: effects on face asymmetry. Comput Biol Med.

[31] McKetin R, McLaren J, Lubman DI, Hides L (2006). The prevalence of psychotic symptoms among methamphetamine users. Addiction.

[32] Brecht ML, Herbeck D (2013). Methamphetamine use and violent behavior: user perceptions and predictors. J Drug Issues.

[33] Noroozi M, Higgs P, Noroozi A (2020). Methamphetamine use and HIV risk behavior among men who inject drugs: causal inference using coarsened exact matching. Harm Reduct J.

[34] Aldana A (2023). Why are some people more vulnerable than others? | Excelerol - America’s #1 brain supplement [Internet]. https://www.excelerol.com/why-are-some-people-more-vulnerable-than-others/.

[35] (2016). The neurobiology of substance use, misuse, and addiction. Facing addiction in America: the surgeon general's report on alcohol, drugs, and health [Internet].

[36] (2023). Novel dopamine D3 receptor antagonist reduces opioid addiction-like behaviors in rats. National Institute on Drug Abuse.

[37] Mutschler NH, Covington HE 3rd, Miczek KA (2001). Repeated self-administered cocaine "binges" in rats: effects on cocaine intake and withdrawal. Psychopharmacology (Berl).

[38] London ED, Kohno M, Morales AM, Ballard ME (2015). Chronic methamphetamine abuse and corticostriatal deficits revealed by neuroimaging. Brain Res.

[39] Koob GF, Volkow ND (2016). Neurobiology of addiction: a neurocircuitry analysis. Lancet Psychiatry.

[40] Parsegian A, See RE (2014). Dysregulation of dopamine and glutamate release in the prefrontal cortex and nucleus accumbens following methamphetamine self-administration and during reinstatement in rats. Neuropsychopharmacology.

[41] Sekine Y, Minabe Y, Kawai M (2002). Metabolite alterations in basal ganglia associated with methamphetamine-related psychiatric symptoms: a proton MRS study. Neuropsychopharmacol.

[42] Chapman DE, Hanson GR, Kesner RP, Keefe KA (2001). Long-term changes in basal ganglia function after a neurotoxic regimen of methamphetamine. J Pharmacol Exp Ther.

[43] Melega WP, Laćan G, Harvey DC, Way BM (2007). Methamphetamine increases basal ganglia iron to levels observed in aging. Neuroreport.

[44] Koob GF (2014). Neurocircuitry of alcohol addiction: synthesis from animal models. Handb Clin Neurol.

[45] Karila L, Weinstein A, Aubin HJ, Benyamina A, Reynaud M, Batki SL (2010). Pharmacological approaches to methamphetamine dependence: a focused review. Br J Clin Pharmacol.

[46] Koob GF, Volkow ND (2010). Neurocircuitry of addiction. Neuropsychopharmacology.

[47] Payer DE, Lieberman MD, London ED (2011). Neural correlates of affect processing and aggression in methamphetamine dependence. Arch Gen Psychiatry.

[48] Lindsey KP, Gatley SJ, Volkow ND (2003). Neuroimaging in drug abuse. Curr Psychiatry Rep.

[49] Feil J, Sheppard D, Fitzgerald PB, Yücel M, Lubman DI, Bradshaw JL (2010). Addiction, compulsive drug seeking, and the role of frontostriatal mechanisms in regulating inhibitory control. Neurosci Biobehav Rev.

[50] Egilmez Y, Jung ME, Lane JD, Emmett-Oglesby MW (1995). Dopamine release during cocaine self-administration in rats: effect of SCH23390. Brain Res.

[51] Sabrini S, Wang GY, Lin JC, Ian JK, Curley LE (2019). Methamphetamine use and cognitive function: a systematic review of neuroimaging research. Drug Alcohol Depend.

[52] Derauf C, Kekatpure M, Neyzi N, Lester B, Kosofsky B (2009). Neuroimaging of children following prenatal drug exposure. Semin Cell Dev Biol.

[53] Haile CN, Varner KJ, Huijing X, Arora R, Orson FM, Kosten TR, Kosten TA (2022). Active and passive immunization with an anti-methamphetamine vaccine attenuates the behavioral and cardiovascular effects of methamphetamine. Vaccines (Basel).

[54] Collins KC, Schlosburg JE, Bremer PT, Janda KD (2016). Methamphetamine vaccines: improvement through hapten design. J Med Chem.

[55] Gentry WB, Rüedi-Bettschen D, Owens SM (2009). Development of active and passive human vaccines to treat methamphetamine addiction. Hum Vaccin.

[56] Stevens MW, Gunnell MG, Tawney R, Owens SM (2016). Optimization of a methamphetamine conjugate vaccine for antibody production in mice. Int Immunopharmacol.

[57] Stevens MW, Rüedi-Bettschen D, Gunnell MG, Tawney R, West CM, Owens SM (2019). Antibody production and pharmacokinetics of METH in rats following vaccination with the METH vaccine, IXT-v100, adjuvanted with GLA-SE. Drug Alcohol Depend.

[58] Schwendt M, Rocha A, See RE, Pacchioni AM, McGinty JF, Kalivas PW (2009). Extended methamphetamine self-administration in rats results in a selective reduction of dopamine transporter levels in the prefrontal cortex and dorsal striatum not accompanied by marked monoaminergic depletion. J Pharmacol Exp Ther.

[59] Truong TT, Kosten TR (2022). Current status of vaccines for substance use disorders: A brief review of human studies. J Neurol Sci.

[60] Arora R, Haile CN, Kosten TA (2019). Preclinical efficacy of an anti-methamphetamine vaccine using E6020 adjuvant. Am J Addict.

[61] Gopalakrishnan A, Richard K, Wahid R, Harley R, Sztein MB, Hawkins LD, Vogel SN (2022). E6020, a TLR4 agonist adjuvant, enhances both antibody titers and isotype switching in response to immunization with hapten-protein antigens and is diminished in mice with TLR4 signaling insufficiency. J Immunol.

[62] Yang F, Kosten TR (2019). Psychopharmacology: neuroimmune signaling in psychiatric disease-developing vaccines against abused drugs using toll-like receptor agonists. Psychopharmacology (Berl).

[63] Laurenzana EM, Byrnes-Blake KA, Milesi-Hallé A, Gentry WB, Williams DK, Owens SM (2003). Use of anti-(+)-methamphetamine monoclonal antibody to significantly alter (+)-methamphetamine and (+)-amphetamine disposition in rats. Drug Metab Dispos.

[64] Everitt BJ (2014). Neural and psychological mechanisms underlying compulsive drug seeking habits and drug memories--indications for novel treatments of addiction. Eur J Neurosci.

[65] Hossain MK, Hassanzadeganroudsari M, Nurgali K, Apostolopoulos V (2020). Vaccine development against methamphetamine drug addiction. Expert Rev Vaccines.

[66] Macur K, Ciborowski P (2021). Immune system and methamphetamine: molecular basis of a relationship. Curr Neuropharmacol.

[67] Papageorgiou M, Raza A, Fraser S, Nurgali K, Apostolopoulos V (2019). Methamphetamine and its immune-modulating effects. Maturitas.

[68] Moszczynska A (2021). Current and emerging treatments for methamphetamine use disorder. Curr Neuropharmacol.

[69] Hossain MK, Hassanzadeganroudsari M, Kypreos E, Feehan J, Apostolopoulos V (2021). Immune to addiction: how immunotherapies can be used to combat methamphetamine addiction. Expert Rev Vaccines.

[70] Zalewska-Kaszubska J (2015). Is immunotherapy an opportunity for effective treatment of drug addiction?. Vaccine.

[71] Jones CM, Houry D, Han B, Baldwin G, Vivolo-Kantor A, Compton WM (2022). Methamphetamine use in the United States: epidemiological update and implications for prevention, treatment, and harm reduction. Ann N Y Acad Sci.

